# Clinical Manifestations of Dengue in Children and Adults in a Hyperendemic Region of Colombia

**DOI:** 10.4269/ajtmh.23-0717

**Published:** 2024-03-19

**Authors:** Jorge Emilio Salazar Flórez, Katerine Marín Velasquez, Ángela María Segura Cardona, Berta Nelly Restrepo Jaramillo, Yuris Esther Ortega Díaz, Luz Stella Giraldo Cardona, Margarita Arboleda Naranjo

**Affiliations:** ^1^ Epidemiology and Biostatistics Group, CES University, Medellín, Colombia;; ^2^ Infectious and Chronic Diseases Study Group (GEINCRO), San Martín University Foundation, Sabaneta, Colombia;; ^3^ Colombian Institute of Tropical Medicine, CES University, Medellín, Colombia

## Abstract

Dengue is the most common arboviral disease in the world. Traditionally, it has affected more adults, but the incidence in children has increased in recent years. Colombia is no stranger to this change; therefore, we aimed to find the differences in signs, symptoms, and clinical, hematological, and hemogram characteristics between children under 12 years old and individuals aged 12 years and older in an endemic region of Colombia in 2020–2022. The analyses were conducted with baseline data, corresponding to a cross-sectional design. Multiple correspondence analysis was used for general, dermatological, and clinical symptom profiles. Discriminant analysis was used for laboratory profiles. Multiple correspondence analysis was applied to nominal categorical data, employing Euclidean distances to analyze age groups. Discriminant analysis was applied to a training sample and validated on a test sample. The overall agreement of the model’s discrimination, sensitivity, specificity, and fit indicators was calculated. The results indicated that individuals under 12 years exhibited distinct dermatological and clinical features, including rash, pruritus, hypotension, lymphocyte count, and platelet count, compared with those aged 12 years and older. In contrast, those 12 years and older were profiled for general and clinical symptoms such as pain (back pain, retro-orbital pain, headache), dizziness, chills, hematuria, tachypnea, and elevated/high hematocrit, hemoglobin, and basophil values. These findings are crucial to understanding the high incidence in children; they also facilitate rapid understanding of the disease in clinical care settings and differentiate it from other febrile outbreaks. This will affect disease control, particularly in severe cases, and reduce mortality.

## INTRODUCTION

Dengue fever, also known as dengue hemorrhagic fever and dengue shock syndrome, is a dynamic, systemic infectious disease caused by female *Aedes* mosquitoes, mainly *Aedes aegypti* and *Aedes albopictus*.^1^ The causative agent of Dengue virus (DENV) is the Flaviviridae family virus with four serotypes of varying virulence.^2^^,^^3^ The WHO’s 2009 classification categorizes it into dengue with warning signs, dengue without warning signs, and severe dengue.^4^ Dengue, the most prevalent arboviral disease, affects 128 countries in tropical and subtropical zones.^2^^,^^5^

Reports from the Pan American Health Organization (PAHO)^6^ show a sharp increase in cases in the Americas, rising from 1.5 million in the 1980s to 16.2 million between 2010 and 2019. Of the 17 million cases reported to the PAHO between 2014 and 2022, South American countries, especially^7^ Brazil and Colombia, reported 84% of cases with the highest severity rates.^7^ Historically, the disease has affected predominantly adults in the Americas, contrasting with patterns in regions such as Asia.^8^ However, the incidence in children has been climbing,^8^^–^^11^ with Brazil and Colombia seeing a significant rise in pediatric cases and dengue-related fatalities since 2008.^9^^–^^11^ By 2023, the disease incidence in children, which was once below 7%, had surged, peaking at a 22.0% average in 2008.^9^ In Colombia, since 2010, half of the severe dengue cases have been in those under 15 years, with one study showing an 85% seroprevalence in children aged 4–11 years.^10^

Symptoms of dengue typically occur 5–7 days after infection, with severe dengue occurring in some cases.^4^ Diagnosis primarily hinges on a distinct fever lasting less than a week, absence of upper respiratory symptoms, and at least two of the following: headache and/or pain behind the eyes, general discomfort, muscle or joint pain, gastrointestinal symptoms such as diarrhea or vomiting, skin rash, petechiae, a positive tourniquet test, leukopenia, thrombocytopenia, or elevated liver enzymes.^4^^,^^5^ These symptoms vary between children and adults.^9^^,^^12^^,^^13^

Previous research indicates that adults with dengue often suffer from myalgia, retro-orbital pain, nausea, and arthralgia, whereas children tend to exhibit vomiting and rashes.^9^^,^^13^ Rapid progression to severe dengue, marked by vascular leakage in children and bleeding in adults, differs between age groups.^14^^,^^15^ Notably, febrile and gastrointestinal symptoms in Bangladeshi children and hepatomegaly and fatigue in Thai infants were observed.^14^^,^^16^^,^^17^ Yet, the distinction between pediatric and adult symptoms remains vague. With dengue’s rise in Latin American children, the need for targeted health interventions has become critical in reducing school absenteeism, hospitalizations, and potential increases in child mortality.^8^^,^^9^

Dengue may be challenging to recognize because of its undifferentiated presentation, with symptoms that are similar to those of other viral illnesses, making differentiation especially difficult in children, who have high exposure to febrile diseases.^9^ In addition, given the absence of specific treatments for dengue, early detection of disease is essential.^18^^,^^19^ The need to identify clear warning signs for early and effective medical response is paramount. In effect, our study aimed to differentiate the signs and symptoms (clinical and hematological) between individuals under 12 years and those 12 years and older in an endemic Colombian region.

## MATERIALS AND METHODS

### Study design and participants.

A cross-sectional study was carried out with patients recruited from 2020 to 2022 in a population of two hyperendemic communities in Antioquia, Colombia: Turbo and Apartadó.^20^ The dengue rate in this region surged by 67.2% from 2015 to 2019, resulting in an 88.9% rise in severe dengue cases^21^ and making the region an important place to study the changing incidence and disease characteristics between children and adults.

Data collection was carried out through active and passive searches in the region’s health centers and in the homes of the participants enrolled in the project. Patients of all ages, sexes, and races with a history of fever of 7 days or less in duration with no apparent focus, accompanied by two or more of the following symptoms were included: headache, retro-ocular pain, myalgias, arthralgia, and rash, with laboratory confirmation of dengue infection. Patients with a history of receiving blood products or under 6 months old were excluded. Patients for this study were recruited and enrolled consecutively according to reports of positive cases in the hospitals of communities of interest in 3 years, from 2020 to 2022.

### Instruments and procedures.

Data were obtained from primary sources by interviewing each patient and from secondary sources by reviewing medical records. Sociodemographic variables included age, sex, race, education, occupation, socioeconomic status, type of social security, and area of origin. Clinical variables, including the type and classification of the disease, the presence and type of comorbidity and co-infection, health care, signs and symptoms, and laboratory variables such as blood count, liver function tests, renal function, and acute phase reactants, were all considered. The clinical and laboratory data analyzed were from the first 5 or 7 days of disease progression, corresponding to the cohort’s baseline.

A group of trained research assistants carried out data collection. This process was carried out by active and passive canvassing of the health centers and participants’ homes. Consent and assent were obtained from all participants prior to administration of the survey and extraction of information from their clinical records.

Venous blood samples were collected from all participating patients using two dry yellow-capped Vacutainer^®^ tubes containing 5–7 mL of separation gel and one (1) purple-capped tube containing EDTA (ethylenediaminetetraacetic acid) anticoagulant. The hospital laboratory was asked to provide blood samples in cases where patients were hospitalized. The diagnosis was confirmed 14–21 days after the onset of symptoms in each patient, during which time a new venous blood sample was taken in a dry tube with a yellow cap and convalescent phase separator gel.

### Diagnosis of DENV infections.

Dengue virus–specific IgM antibodies were determined by the Panbio^®^ capture ELISA (Baltimore, MD); this procedure was performed on all acute and convalescent serum samples. Dengue virus–specific IgG antibodies were determined by the Focus^®^ (Focus Diagnostics Inc. Cypress, CA) capture ELISA method; this procedure was performed on all acute-phase serum samples, and only those samples that tested negative for this test in the acute-phase were repeated in the convalescent phase. Dengue virus NS1 antigen was measured using the Panbio capture ELISA; this procedure was performed on all acute-phase serum samples. Viral RNA was detected using the CDC Trioplex diagnostic kit with the reverse transcription polymerase chain reaction (RT-PCR) method; this test was performed on all acute-phase serum samples after RNA extraction. The DENV serotype was identified by detection of viral RNA by RT-PCR using a CDC DENV serotype detection kit; this test was used on all acute-phase specimens that tested positive for dengue in the CDC Trioplex. Laboratory test results were reported to all participants.

This study also diagnosed co-infection with Leptospira, Zika, and Chikungunya. Leptospirosis was detected in all samples using the commercially available Panbio IgM capture antibody ELISA and the Colombian Institute of Tropical Medicine–standardized microscopic agglutination test confirmatory test, which allows identification of serovars and antibody titers. For chikungunya, all acute-phase serum samples were tested for IgM antibodies using the Novatec (Novatec Immundiagnostica GmbH, Dietzenbach, Germany)^®^ capture ELISA according to the manufacturer’s instructions and for viral RNA using the RT-PCR technique with a CDC Trioplex diagnostic kit. Finally, Zika was tested in acute-phase serum samples by RT-PCR detection of viral RNA using a CDC Trioplex diagnostic kit.

## STATISTICAL ANALYSES

Despite the 3-year recruitment period, the analyses used data from the time of diagnosis or confirmation, representing a cross-sectional analysis. To describe the population, both absolute and relative frequencies were examined. Dengue proportions were estimated according to severity: severe dengue, dengue with warning signs, and dengue without warning signs. The presence of primary and secondary infection and co-infection was analyzed. To assess potential differences in dengue presentation between children and adults, the population was segmented into two age groups: 1) under 12 years old and 2) 12 years or older. All subsequent analyses focused on this age group variable. The population was divided into these groups to investigate differences between children and other age groups, considering the documented increase in dengue incidence among Latin American children in previous studies.^9^^,^^22^^–^^24^ In Colombia, according to Article 3 of the Childhood and Adolescence Code, a child is defined as an individual under 12 years of age.^25^ Therefore, the age of 12 was used as a criterion for categorizing the groups of interest in this study. Individuals under 12 years of age will be referred to as “children,” whereas participants aged 12 years and older will be generally referred to as “adults,” although not all individuals in this group are legally considered adults, as the legal definition in Colombia typically starts at 18 years of age. The primary objective was to distinguish children as a distinct group from individuals of other age groups, rather than basing it on legal definitions of adulthood. Similar age groupings have been used in previous literature in this country and region, demonstrating significant changes in dengue incidence at this age.^10^^,^^26^ Furthermore, these studies also differentiated between children (ranging from 11 to 15 years) and other age groups. The comparability of immunological responses in individuals aged 11–15 years is based on key developmental changes in their immune systems. During these years, significant maturation and adaptation occur, mirroring adult immune responses. This includes thymic involution (the transformation of the thymus during puberty), leading to a more adultlike composition of T cells, and an increase in both antibody responses and B cell repertoire diversity. Despite some differences, the overall immune response in this age group is sufficiently similar to that of adults, justifying their comparison in our study. This underpins our decision to categorize those under 12 years and those older than 12 years as distinct groups.

The χ^2^ test or Fisher test was used to investigate the association between sociodemographic, clinical, and laboratory factors about population group membership (under 12 years versus 12 years and older). A *P-*value of less than 0.05 was considered statistically significant. Discriminant and multiple correspondence analyses were used to define the clinical profile of the disease in children.

Multiple correspondence analysis (MCA) was used to establish a profile of clinical and hematological findings based on the age groups as interest clusters. Multiple correspondence analysis was applied to nominal categorical data, employing Euclidean distances to analyze age groups. Pairwise data were tabulated in a K-by-K table and were visually graphed on two-dimensional graphs. The variance of the two estimated dimensions was calculated. Multiple MCAs were performed to explore differences between groups.

The differentiation profile of the hemogram according to age group was investigated using discriminant analysis. Discriminant analysis classified participants by age into 1) those under 12 years old and 2) those 12 years old or older. Discriminant variables were derived from baseline laboratory tests: hematocrit, hemoglobin, platelets, neutrophils, lymphocytes, leukocytes, eosinophils, monocytes, and basophils. Participants were randomly split into two groups for training (70%) and testing (30%). A linear discriminant model (LDA) was examined on the training dataset and subsequently executed on the test dataset to ascertain group membership.

Discriminant variables were assessed for multivariate normality. The M Box test was performed to analyze the independence assumption of the covariances. The initial variables failed to meet the necessary assumptions and underwent standardization using Box-Cox to obtain a more suitable discriminant function. The model’s discriminatory capability was assessed by the percentage of correct classifications in each group (classification matrix). Wilk’s λ parameter was set to determine the final model’s discriminatory power. In addition, the model’s canonical correlation (which ranges from −1 to +1) was examined to support its efficiency. Furthermore, the following metrics were used to assess the discriminative ability of the resulting function: accuracy, error, positive predictive value, negative predictive value, sensitivity, and specificity.

Analyses were performed in open-source software R v. 4.2.3. The FactoMineR and Factoextra libraries were used for MCA. For discriminant analysis, the LDA function of the MASS package was used.

### Ethics considerations.

This study adhered to the principles and guidelines established in the Declaration of Helsinki^27^ and Colombian Resolution 8430 of 1993.^28^ The project is classified as having higher than minimal risk because of the blood sampling process. The ethical component of the project was approved by the Bioethics Committee of the Colombian Institute of Tropical Medicine (CES) through Act 66, dated June 14, 2019.

## RESULTS

### Participants’ characteristics and differences in signs and symptoms of dengue.

A total of 192 patients were included in the study. Of these, 111 were children under 12 years, and 81 were 12 years or older. Just over half of the participants had dengue with alarming signs (58.3%), with similar proportions in both age groups. Severe dengue cases were low, but the proportion in children under 12 years (4.5%) was almost 4 times higher than in older children. Primary dengue was more common in children under 12 years of age (11.7%), whereas secondary dengue was more common in adults (90.1%), but there were no significant statistical differences. There were also no cases of co-infection with Zika and Chikungunya. A total of 19 cases of co-infection with leptospirosis were observed, with no significant differences between age groups (Table 1).

**Table 1:** Demographics, signs, symptoms, and clinical differences between age groups

Variables	Under 12 Years Old (*n* = 111)		12 Years Old or Older (*n* = 81)		Total (*n* = 192)	*P-*Value*
	*n*	%	*n*	%	*N*	
Demographic variables						
Sex						
Female	56	50.5	52	64.2	108	0.058
Male	55	49.5	29	35.8	84	–
Area of residence						
Rural	44	39.6	39	48.1	83	0.240
Urban	67	60.4	42	51.9	109	–
Type of infection						
Primary	13	11.7	8	9.9	21	0.687
Secondary	98	88.3	73	90.1	171	–
Clinical form						
Severe dengue	5	4.5	1	1.2	6	0.372^†^
Dengue with warning signs	65	58.6	47	58.0	112	–
Dengue without warning signs	41	36.9	33	40.7	74	–
Co-infection						
Leptospirosis co-infection	9	8.1	10	12.3	19	0.331
General symptoms						
Fever	100	100.0	75	92.6	186	**0.004**
Myalgia	90	81.1	69	85.2	159	0.457
Arthralgia	85	76.6	69	85.2	154	0.139
Adynamia	91	82.0	66	81.5	157	0.929
Asthenia	78	70.3	54	66.7	132	0.595
Headache	89	80.2	75	92.6	164	**0.016**
Retro-orbital pain	44	39.6	45	55.6	89	**0.029**
Back pain	8	7.2	28	34.6	36	**0.000**
Dizziness	35	31.5	38	46.9	73	**0.030**
Chills	58	52.3	55	67.9	113	**0.030**
Vomiting	71	64.0	44	54.3	115	0.178
Abdominal pain	75	67.6	52	64.2	127	0.626
Diarrhea	39	35.1	27	33.3	66	0.795
Dermatological signs and symptoms						
Itching	62	55.9	32	39.5	94	**0.025**
Rash	24	21.6	8	9.9	32	**0.031**
Desquamation	4	3.6	2	2.5	6	1.000
Clinical and hematological findings						
Hematuria	5	4.5	14	17.3	19	**0.003**
Hypotension	63	56.8	16	19.8	79	**0.000**
Tachypnea	53	47.7	56	69.1	109	**0.003**
Bradycardia	19	17.1	14	17.3	33	0.976
Thrombocytopenia^‡^	72	64.9	51	63.0	123	0.119
Hemoconcentration (>10%)^‡^	25	22.5	25	30.9	50	0.082

* χ^2^ of association.

^†^ Fisher test.

^‡^ Missing: 5.

Bold value denotes the statistical significance.

Table 1 shows the demographics, signs, symptoms, and differences between age groups. Males and urban residents represented higher proportions in children under 12 years of age, whereas females represented the highest proportions in those aged 12 years and older. In both age groups, 58.0% of dengue cases were reported with alarming symptoms, but no statistical association existed. Significant differences in the prevalence of common symptoms, such as fever, headache, retro-orbital pain, back pain, dizziness, and chills (*P-*value <0.05), were observed among the age groups. The group aged 12 years or older had a higher prevalence of these symptoms, except for fever, which was more prevalent in those younger than 12 years (100%). Regarding dermatological signs and symptoms, significant differences were observed for itching and rash (*P-*value <0.05). The prevalence of these symptoms was higher in the group of children under 12 years of age, doubling the prevalence found in those older than 12 years of age. Lastly, clinical and hematological findings revealed statistically significant differences in hematuria, hypotension, and tachypnea (*P-*value <0.05). However, it was only in the case of hypotension that the group under 12 years of age exhibited a prevalence exceeding that of those 12 years of age and older by more than half.

Table 2 shows the difference in hemogram between age groups. The groups’ hematocrit, hemoglobin, platelet, lymphocyte, and basophil values were statistically different (*P-*value <0.05). Platelets and the percentage of lymphocytes had higher mean values in children younger than 12 years. In contrast, hematocrit, hemoglobin, and percentage of basophils had higher mean values in participants 12 years and older.

**Table 2:** Difference in hemogram profile by age group

Variables	Under 12 Years Old (*n* = 111)		12 Years Old or Older (*n* = 81)		*P*-Value
	Min–Max	Mean (SD)	Min–Max	Mean (SD)	
Hematocrit (%)	28.9–43.7	35.9 (3.3)	23.0–48.3	38.8 (5.1)	**0.000** *
Hemoglobin	8.7–16.1	12.0 (1.3)	7.6–16.8	13.0 (1.8)	**0.000** *
Platelets	24,000–534,000	184,867.9 (112,270.3)	15,000–376,000	143,271.6 (84,038.9)	**0.006** ^†^
Leukocytes	1,437–35,580	5,928.7 (4,636.5)	1,560–45,000	5,365.2 (4,969.5)	0.427^†^
Neutrophils (%)	5.0–90.2	47.7 (21.5)	4.8–92.0	52.0 (20.2)	0.167^†^
Lymphocytes (%)	3.9–81.2	39.9 (19.2)	5.8–68.2	30.7 (14.5)	**0.001** *
Eosinophils (%)	0.0–15.0	2.7 (3.4)	0.0–19.7	4.0 (4.9)	0.064^†^
Monocytes (%)	0.4–38.8	8.9 (5.6)	0.6 (31.9)	10.7 (5.9)	0.067^†^
Basophils (%)	0.0–3.5	1.0 (0.7)	0.0–5.0	1.3 (1.1)	**0.041** ^†^

Max = maximum; Min = minimum.

* Mann-Whitney *U* test.

^†^ Student’s *t*-test.

Bold value denotes the statistical significance.

### Dengue signs and symptoms: Profile by age group.

**Figure 1.**
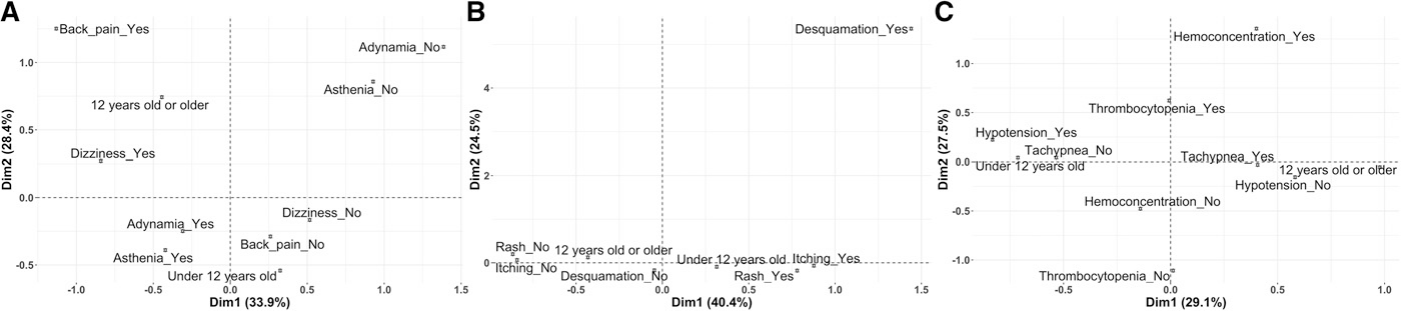
Profiles in children under and over 12 years of age relating to general symptoms, dermatological profile, and clinical findings. (**A**) General signs and symptoms profiled by age group. (**B**) Dermatological signs and symptoms profiled by age group. (**C**) Hematological signs and symptoms profiled by age group. Dim = dimension.

Figure 1A shows the profile of age groups according to general symptoms. Dimension 1 explained 33.9% of the variance. By proximity, it was clear that the cluster of children under 12 years of age was represented by the absence of back pain and dizziness, whereas those older than this age group were profiled by the presence of both situations.

Dimension 1 explained 40.4% of the group variability for dermatological signs and symptoms. Itching and rash explained the profile of children under 12 years of age. In contrast, these dermatological conditions were absent in those 12 years and older (Figure 1B).

Finally, dimension 1 explained 29.1% of the variability in the clinical and hematological findings. The group younger than 12 years was characterized by hypotension and the absence of tachypnea. In contrast, those aged 12 years and older did not present with hypotension but were represented by tachypnea (Figure 1C).

The training set included 70% of randomly chosen records, with the remaining 30% comprising the test set. Eighty individuals were included in the first sample for discriminant analysis training, with 38 subjects remaining in the test sample. In the discriminant analysis, the variables that distinguished children under 12 years from older age groups were hematocrit, platelets, lymphocytes, eosinophils, monocytes, and basophils. The confusion matrix indicates that 89.5% of cases initially grouped by age were accurately classified in the final test model, with a sensitivity of 87.5% and specificity of 90.9%. The high canonical correlation value (0.77) supports the appropriate classification of patients into the age groups previously determined by the theoretical criteria outlined in the methodology (Table 3).

**Table 3:** Goodness of fit indicators in discriminant analysis by age group according to hemogram profile

Indicators	Training Set (70%)	Test Set (30%)
Accuracy (%)	76.3	89.5
Error (%)	23.7	10.5
PPV (%)	82.2	87.5
NPV (%)	68.6	90.9
Sensitivity (%)	77.1	87.5
Specificity (%)	75.0	90.9
Canonical correlation	0.57	0.77
Eigenvalue	0.47	1.45
Wilks λ	0.68 *P-*value <0.001	0.41 *P-*value <0.001

PPV = positive predictive value; NPV = negative predictive value. Training set: Mbox:0.024; Mardia mSkewness: 0.018; Mardia mKurtosis: 0.337; Henze-Zirkler: 0.088; and Doornik-Hansen: 0.248. Test set: Mbox:0.174; Mardia mSkewness: 0.152; Mardia mKurtosis: 0.987; Henze-Zirkler: 0.010; and Doornik-Hansen: 0.041.

## DISCUSSION

In our study, the cases of dengue with alarm signs were similar in individuals under 12 years (58.6%) and those over 12 years (58.0%); however, severe dengue in children (4.5%) exceeded that in adults. Children and adults appear to differ in clinical presentation and severity of infection. The findings indicate that those under 12 years typically exhibited dermatological symptoms (rash, itching) and some clinical signs (hypotension) and fever in their profiles. However, those aged 12 years and older were predominantly characterized by general signs and symptoms (headache, retro-orbital pain, back pain, dizziness, chills) and some clinical signs (hematuria, tachypnea). Hemogram analysis also identified distinguishing factors among age groups. Children under 12 years had higher lymphocyte and platelet counts, whereas those older than 12 had higher hematocrit, hemoglobin, and basophil levels. Monocytes and eosinophils had no significant differences between the groups, although both were included in the discriminant model.

Childhood dengue incidence has undergone a significant rise in the Americas, particularly in countries such as Mexico, Honduras, and Colombia, where seroprevalence rates have been on the rise, indicating a growing disease burden among children.^7^^,^^9^^–^^11^^,^^23^^,^^29^^,^^30^ Brazil also experienced high child hospitalization rates during epidemics in 2007, 2008, and 2010.^11^ In 2023, Peru reported around 46,000 dengue cases, including numerous child fatalities, whereas Puerto Rico revealed seroprevalence rates of 9% in younger children and 44% in those aged 9–16 years.^23^^,^^31^ In the present study, over a 3-year period from 2020 to 2022, we identified 192 dengue cases in the region. It is crucial to note that this time frame coincided with the COVID-19 pandemic, making it challenging to differentiate between the two diseases owing to overlapping respiratory symptoms.^32^ As a result, many dengue cases were likely misclassified as COVID-19 or went unreported, contributing to underreporting.

The rise in childhood dengue cases in the region may be due to new genotypes, with all four dengue serotypes present instead of just one or two.^11^^,^^33^ For instance, the detection of Southeast Asia’s DENV-2 lineage II in Brazil and the cosmopolitan genotype in Peru and Brazil in 2019 and 2021 align with increased pediatric hospitalizations, similar to trends in Asia.^8^^,^^11^^,^^33^ In addition, factors such as increased rainfall and climate change may heighten dengue risk in tropical regions worldwide.^34^^,^^35^

A multivariate analysis shows clear differences in dengue symptoms between adults and children: Adults often suffer from myalgia, eye pain, nausea, and joint pain, whereas children are prone to vomiting and rashes.^9^ A possible explanation for this variation is the recent rise in dengue symptoms among children, leading to an increase in medical diagnoses for this demographic, a contrast to earlier trends.^24^^,^^29^ The unique clinical presentation in children highlights how their responses to dengue, particularly symptomatic forms, differ from those of adults and may be due to their lack of immunity to various dengue serotypes, making them more susceptible.^23^ These findings, especially the symptom differences observed in children under and over 12 years old, could shed light on age-specific responses to dengue.

In our study, children under 12 years often exhibited rash, itching, fever, hypotension, and elevated lymphocytes and platelets, whereas older individuals commonly showed localized pains, dizziness, chills, hematuria, tachypnea, and increased hematocrit and hemoglobin levels. Fever was notably universal among the younger group, a finding echoed by multiple studies.^22^^–^^24^ Our data did not reflect the commonly reported gastrointestinal symptoms in young dengue patients.^23^ A 2023 Argentinian study found headaches in 75% of 1- to 15-year-olds, with 45% also reporting rash and abdominal pain.^22^ Our under–12-year-old participants had a 22% incidence of rash.

Distinct symptoms in children are crucial for detecting severe dengue, increasingly prevalent in younger demographics.^24^ A 2022 Colombian study found a 45.1% incidence of severe dengue in children aged 4–9 years.^24^ In line with global observations, older subjects in our research presented with milder symptoms. The 2019 Bangladesh outbreak showed adults experienced more gastrointestinal and plasma leakage issues, differing from the hemorrhagic tendencies of past outbreaks.^36^ Reports from Latin America indicate that fever, myalgias, intense abdominal pain, and vomiting are common in pediatric dengue cases, along with a drop in platelet levels.^22^^,^^24^ Our findings of fever, hypotension, unique lymphocyte and platelet counts, rash, and itching in children underpin the critical need for prompt clinical assessment due to the strong association with severe dengue.^4^^,^^22^^,^^37^

A 2023 study evaluated the diagnostic accuracy of 985 dengue cases in Puerto Rico using 2015 PAHO guidelines, with a focus on age-related impacts.^13^ Findings revealed that children under 5 years were more prone to rashes and petechiae, whereas adults more commonly suffered from aches and headaches. To enhance diagnostic specificity, the study recommended requiring at least two symptoms from vomiting, petechiae, rash, or leukopenia (specificity 68%, sensitivity 71%) or using PAHO criteria with additional tests for aspartate aminotransferase (AST) levels, platelet count, skin itching, and absence of cough (specificity 51%, sensitivity 82%).^13^ These criteria align with our study’s findings for children under and over 12 years.

Internationally, common symptoms among those under 18 years are fever, body aches, headaches, and rash, accompanied by increased hematocrit, leukopenia, and thrombocytopenia.^12^^,^^38^ In India, dengue in children under 12 years typically involves narrow pulse pressure, mucosal bleeding, and third space fluid loss.^39^ Fever, headache, muscle pain, nausea, and vomiting are widespread across different regions and ages, with thrombocytopenia, anemia, and leukopenia as standard blood findings.^40^^,^^41^ High hematocrit levels (>20% from baseline) and low platelet counts (<50,000/mm^3^) have been flagged as severe dengue indicators.^16^ A review linked severe dengue in adults to comorbidities, reinfections, and lower starting platelet counts.^8^ Corroborating this, prior meta-analyses have deduced that swift shifts in platelet count and AST levels within the initial 72 hours after fever onset can forecast severe cases.^42^ However, in cases of severe dengue, there is also a significant number of infants with decreased platelets and serious organ damage.^24^ In effect, our study points to platelet count as a critical indicator in children under 12 years.

Prompt detection of dengue is critical in the studied cities, where it often mimics other febrile illnesses, leading to mismanagement. In Asia, undifferentiated febrile illnesses have resulted in a dengue fatality rate close to 25%.^43^ Likewise, in Caribbean children, Dengue, Chikungunya, and Zika have contributed to significant attributable morbidity and mortality.^44^ Diagnosis is further complicated by clinical similarities and the limited availability and expense of confirmatory testing.^43^ Moreover, early diagnosis or management of suspected cases can prevent the use of inappropriate treatments, such as antibiotics, which could result in grave outcomes or fatalities.^45^

The present study has some limitations. First, the profiles were generated using the baseline of the study, so the fluctuating nature of the risk factors was not considered. Second, patients were recruited from a specific geographic area. However, Colombia is hyperendemic, and this region is one of the most representative places of the outbreak. Third, we analyzed only hospitalized patients or cases identified by community surveillance, which does not represent all the patients with dengue in this region. Fourth, patients with febrile illness and at least two related symptoms were included, but the co-circulation of other febrile illnesses such as Zika and chikungunya in the region made using only fever as a criterion operationally impractical. To mitigate this, the study used both community and hospital searches to minimize the exclusion of significant cases. Fifth, it is worth noting that the lack of information about a patient’s comorbidities and the lack of some laboratory data were limitations of this study. Sixth, we also discovered a noteworthy loss of data in complete blood counts. As a result, the discriminant analyses were executed on a reduced sample. Finally, although the discriminant analysis showed differences in laboratory tests between children and adults, this needs to be further investigated to establish the relationship with disease progression. This was not explored in depth because of the low proportion of severe dengue cases in the study, which may also be explained by underdiagnosis due to the presence of the COVID-19 pandemic.

Despite the acknowledged constraints, this study provides foundational data regarding clinical and hematological characteristics of dengue patients. Furthermore, it provides corroborating evidence on the clinical and laboratory aspects that can be used to profile the key interest groups. Among its strengths, it is also important to mention that this study represents a unique cohort built up over 3 years. Confirmed diagnosis was ensured 14–21 days after enrollment. Despite the pandemic context, community case finding was carried out. In addition, the study ensured follow-up of patients until disease resolution.

Our study holds significance for clinical and primary healthcare, which serves as the link to healthcare facilities, to facilitate precise presumptive identification of dengue and enhance attention toward early warning signals in children, resulting in a reduction in case severity or mortality. Although dengue care guidelines are available to aid in the clinical recognition of symptoms, changes in dengue serotypes during outbreaks and the possibility of reinfection may result in altered manifestations of infection. Thus, it is important to update patient profiles, particularly in populations such as children, who presently assume a pivotal role in outbreaks across the Americas. Hence, upholding evidence-based medical practice and care remains crucial. The present study provides valuable insights for future researchers on the conduct of similar studies across various care levels.

## CONCLUSION

Understanding and recognizing the clinical and laboratory changes that occur during dengue fever can help in devising an effective approach and reducing the chances of severe clinical manifestations in children. Dermatological and clinical symptoms, including rash, itching, hypotension, lymphocytes, and platelets, were frequent in children younger than 12 years. Meanwhile, individuals aged 12 years and older exhibited general and clinical signs and symptoms, including headache, retro-orbital pain, back pain, dizziness, chills, hematuria, tachypnea, and elevated levels of hematocrit, hemoglobin, and basophils. In fact, these findings could prove crucial for primary healthcare, healthcare centers, and healthcare professionals in identifying distinctive warning cases of dengue fever in children and adults, as well as distinguishing cases from other febrile outbreaks.

## References

[1] Huang C Tsai Y Wang S Wang W Chen Y, 2021. Dengue vaccine: An update. Expert Rev Anti Infect Ther 19: 1495–1502. 34182875 10.1080/14787210.2021.1949983

[2] Salles TS , 2018. History, epidemiology and diagnostics of dengue in the American and Brazilian contexts: A review. Parasit Vectors 11: 264. 29690895 10.1186/s13071-018-2830-8 PMC5937836

[3] de Lourdes Muñoz M Límon-Camacho G Tovar R Diaz-Badillo A Mendoza-Hernández G Black WC 4th, 2013. Proteomic identification of dengue virus binding proteins in *Aedes aegypti* mosquitoes and *Aedes albopictus* cells. BioMed Res Int 2013: 875958. 24324976 10.1155/2013/875958 PMC3842078

[4] World Health Organization , 2009. *Dengue Guidelines for Diagnosis, Treatment, Prevention and Control: New Edition*. Geneva, Switzerland: WHO. Available at: https://www.who.int/publications/i/item/9789241547871. Accessed September 14, 2023. 23762963

[5] Bhatt P Sabeena SP Varma M Arunkumar G, 2021. Current understanding of the pathogenesis of dengue virus infection. Curr Microbiol 78: 17–32. 33231723 10.1007/s00284-020-02284-w PMC7815537

[6] Pan American Health Organization , 2020. Integrated Management Strategy for Arboviral Disease Prevention and Control in the Americas. Washington, DC: Pan American Health Organization.

[7] Pan American Health Organization , 2022. Reported Cases of Dengue Fever in the Americas. Washington, DC: Pan American Health Organization.

[8] Wakimoto MD Camacho LA Guaraldo L Damasceno LS Brasil P, 2015. Dengue in children: A systematic review of clinical and laboratory factors associated with severity. Expert Rev Anti Infect Ther 13: 1441–1456. 26536064 10.1586/14787210.2015.1100534

[9] Fonseca SNS, 2023. Changing epidemiology of dengue fever in children in South America. Curr Opin Pediatr 35: 147–154. 36715049 10.1097/MOP.0000000000001220

[10] Velandia-Romero ML , 2020. Prevalence of dengue antibodies in healthy children and adults in different Colombian endemic areas. Int J Infect Dis 91: 9–16. 31733358 10.1016/j.ijid.2019.10.045

[11] Wunderlich J Acuna-Soto R Alonso WJ, 2018. Dengue hospitalisations in Brazil: Annual wave from west to east and recent increase among children. Epidemiol Infect 146: 236–245. 29235427 10.1017/S0950268817002801 PMC9148759

[12] Tran XD , 2023. Aetiology of acute undifferentiated fever among children under the age of five in Vietnam: A prospective study. J Epidemiol Glob Health 13: 163–172. 37258852 10.1007/s44197-023-00121-4 PMC10231849

[13] Odio CD Sánchez-González L Delorey M Adams LE Jones ES Lorenzi O Munoz-Jordan J Rivera-Amill V Paz-Bailey G, 2023. The effect of age on dengue presentation and the diagnostic accuracy of the 2015 Pan American Health Organization case criteria in a Puerto Rican cohort. Open Forum Infect Dis 10: ofad373. 37663092 10.1093/ofid/ofad373 PMC10468746

[14] Afroze S Shakur S Wahab A, 2019. Clinical profile of dengue and predictors of its severity among children. Am J Pediatr 5: 219–223.

[15] Rosenberger K Alexander N Martínez E Lum L Dempfle C Junghanns T Wills B Jaenisch T; DENCO Clinical Study Group , 2020. Severe dengue categories as research endpoints: Results from a prospective observational study in hospitalised dengue patients. PLoS Negl Trop Dis 14: e0008076. 32130212 10.1371/journal.pntd.0008076 PMC7055818

[16] Khan MAS , 2021. Clinical spectrum and predictors of severity of dengue among children in 2019 outbreak: A multicenter hospital-based study in Bangladesh. BMC Pediatr 21: 478. 34715835 10.1186/s12887-021-02947-y PMC8555185

[17] Prommalikit O Thisyakorn U Thisyakorn C, 2021. Clinical manifestations of early childhood dengue virus infection in Thailand. Med J Malaysia 76: 853–856. 34806672

[18] Lee LK Earnest A Carrasco LR Thein TL Gan VC Lee VJ Lye DC Leo Y-S, 2013. Safety and cost savings of reducing adult dengue hospitalization in a tertiary care hospital in Singapore. Trans R Soc Trop Med Hyg 107: 37–42. 23296696 10.1093/trstmh/trs009 PMC4023275

[19] Lee H Hyun S Park S, 2023. Comprehensive analysis of multivariable models for predicting severe dengue prognosis: Systematic review and meta analysis. Trans R Soc Trop Med Hyg 117: 149–160. 36445309 10.1093/trstmh/trac108

[20] Cámara de Comercio de Medellín , 2015. Perfil Socioeconómico de la Subregión del Urabá. Medellín. Available at: https://www.scribd.com/document/468160191/Perfil-socio-economico-Uraba. Accessed September 17, 2023.

[21] Secretaría Seccional de Salud y Protección Social de Antioquia , 2020. *Eventos en Salud Pública*. Available at: https://dssa.gov.co/index.php/vigilancia-en-salud-publica. Accessed September 20, 2023.

[22] Fiora MB Gonzalvez ML Aguirre JP Bacigalupo A Garnero A Rosa AM Obrador MD Grecco C, 2023. Observational study of clinical, epidemiological, and laboratory characteristics of pediatric patients with dengue in the city of Cordoba. Arch Argent Pediatr 122: e202202972. 37639337 10.5546/aap.2022-02972.eng

[23] Sanjeet B, 2023. Dengue outbreak in Peru affects adults and children. Lancet Infect Dis 23: e339. 37633296 10.1016/S1473-3099(23)00229-3

[24] Ricardo-Rivera SM , 2022. Mapping dengue in children in a Colombian Caribbean region: Clinical and epidemiological analysis of more than 3500 cases. Infez Med 30: 602–609. 36482961 10.53854/liim-3004-16 PMC9715006

[25] Ministerio de la Protección Social, Instituto Colombiano de Bienestar Familiar , 2006. *Ley 1098 de 2006: Código de la Infancia y la Adolescencia*. Avaible at: https://www.fiscalia.gov.co/colombia/wp-content/uploads/2012/01/Ley-1098-de-2006.pdf. September 17, 2023.

[26] Hammon S Balmaseda A Pérez L Téllez Y Saborio S Mercado J, 2005. Difference in dengue severity in infants, children, and adults in a 3-year hospital based study in Nicaragua. Am J Trop Med Hyg 73: 1023–1070. 16354813

[27] Asociación Médica Mundial , 2019. Declaración de Helsinki de la AMM-Principios Eticos para las Investigaciones Médicas en Seres Humanos. 64^a^ Asamblea General. Fortaleza, Brazil: AMM.

[28] República de Colombia, Ministerio de Salud , 1993. *Resolución 8430 de 1993: Por la Cuál se Establecen las Normas Científicas, Técnicas y administrativas para la Investigación en Salud*. Available at: https://www.minsalud.gov.co/sites/rid/Lists/BibliotecaDigital/RIDE/DE/DIJ/RESOLUCION-8430-DE-1993.PDF. Accessed September 20, 2023.

[29] L’Azou M Moureau A Sarti E Nealon J Zambrano B Wartel TA Villar L Capeding MR Ochiai RL; CYD14 Primary Study Group, CYD15 Primary Study Group , 2016. Symptomatic dengue in children in 10 Asian and Latin American countries. N Engl J Med 374: 1155–1166. 27007959 10.1056/NEJMoa1503877

[30] Malavige GN Jeewandara C Ghouse A Somathilake G Tissera H, 2021. Changing epidemiology of dengue in Sri Lanka: Challenges for the future. PLoS Negl Trop Dis 15: e0009624. 34411101 10.1371/journal.pntd.0009624 PMC8375976

[31] Adams LE Hitchings MDT Medina FA Rodriguez DM Sánchez-González L Moore H Whitehead SS Muñoz-Jordán JL Rivera-Amill V Paz-Bailey G, 2023. Previous dengue infection among children in Puerto Rico and implications for dengue vaccine implementation. Am J Trop Med Hyg 109: 413–419. 37308104 10.4269/ajtmh.23-0091 PMC10397428

[32] Nicolete VC , 2021. Interacting epidemics in Amazonian Brazil: Prior dengue infection associated with increased coronavirus disease 2019 (COVID-19) risk in a population-based cohort study. Clin Infect Dis 73: 2045–2054. 33956939 10.1093/cid/ciab410 PMC8135953

[33] Amorim MT , 2023. Emergence of a new strain of DENV-2 in South America: Introduction of the cosmopolitan genotype through the Brazilian-Peruvian border. Trop Med Infect Dis 8: 325. 37368743 10.3390/tropicalmed8060325 PMC10305074

[34] Wang Y , 2023. Impact of climate change on dengue fever epidemics in South and Southeast Asian settings: A modelling study. Infect Dis Model 8: 645–655. 37440763 10.1016/j.idm.2023.05.008 PMC10333599

[35] Gutierrez JA Laneri K Aparicio JP Sibona GJ, 2022. Meteorological indicators of dengue epidemics in non-endemic northwest Argentina. Infect Dis Model 7: 823–834. 36474869 10.1016/j.idm.2022.10.004 PMC9709237

[36] Yesmin S Sarmin S Ahammad AM Rafi MA Hasan MJ, 2023. Epidemiological investigation of the 2019 dengue outbreak in Dhaka, Bangladesh. J Trop Med 2023: 8898453. 36968192 10.1155/2023/8898453 PMC10036172

[37] Rey L Villa L, 2012. Linfocitos atípicos en dengue: Papel en el diagnóstico y pronóstico de la enfermedad: Revisión sistemática de la literatura. Rev Cienc Salud 10: 323–325.

[38] Islam S Khan MAS Badal MFA Khan MZI Gozal D Hasan MJ, 2022. Clinical and hematological profiles of children with dengue residing in a non-endemic zone of Bangladesh. PLoS Negl Trop Dis 16: e0010847. 36215330 10.1371/journal.pntd.0010847 PMC9584401

[39] Gayathri V Lakshmi SV Murugan SS Poovazhagi V Kalpana S, 2023. Development and validation of a bedside dengue severity score for predicting severe dengue in children. Indian Pediatr 60: 359–363. 36757000 10.1007/s13312-023-2880-7 PMC10185942

[40] Ferede G Tiruneh M Abate E Wondimeneh Y Gadisa E Howe H Aseffa A Tessema B, 2018. A study of clinical, hematological, and biochemical profiles of patients with dengue viral infections in northwest Ethiopia: Implications for patient management. BMC Infect Dis 18: 616. 30514223 10.1186/s12879-018-3557-z PMC6278031

[41] Rafi A Mousumi AN Ahmed R Chowdhury RH Wadood A Hossain G, 2020. Dengue epidemic in a non-endemic zone of Bangladesh: Clinical and laboratory profiles of patients. PLoS Negl Trop Dis 14: e0008567. 33048921 10.1371/journal.pntd.0008567 PMC7553334

[42] Thach TQ , 2021. Predictive markers for the early prognosis of dengue severity: A systematic review and meta-analysis. PLoS Negl Trop Dis 15: e0009808. 34610027 10.1371/journal.pntd.0009808 PMC8519480

[43] Morch K , 2023. Clinical features and risk factors for death in acute undifferentiated fever: A prospective observational study in rural community hospitals in six states of India. Trans R Soc Trop Med Hyg 117: 91–101. 36130240 10.1093/trstmh/trac091 PMC9890314

[44] Christie CDC Lue AM Melbourne-Chambers RH, 2023. Dengue, chikungunya and Zika arbovirus infections in Caribbean children. Curr Opin Pediatr 35: 155–165. 36801979 10.1097/MOP.0000000000001229 PMC10090388

[45] Robinson ML , 2018. Antibiotic utilization and the role of suspected and diagnosed mosquito-borne illness among adults and children with acute febrile illness in Pune, India. Clin Infect Dis 66: 1602–1609. 29211830 10.1093/cid/cix1059 PMC5930254

